# Predictors and characteristics of Rib fracture following SBRT for lung tumors

**DOI:** 10.1186/s12885-023-10776-8

**Published:** 2023-04-12

**Authors:** Michael P. Carducci, Baskaran Sundaram, Benjamin A. Greenberger, Maria Werner-Wasik, Gregory C. Kane

**Affiliations:** 1 Department of Medicine, Sidney Kimmel Medical College at Thomas Jefferson University, 1025 Walnut St, suite 840, 19107 Philadelphia, PA USA; 2 Department of Radiology, Thomas Jefferson University Hospital, 132 South 10th St, Floor 10, 19107 Philadelphia, PA USA; 3 Department of Radiation Oncology, Sidney Kimmel Cancer Center at Thomas Jefferson University, 111 South 11th St Suite G-301, 19107 Philadelphia, PA USA; 4 Department of Medicine, Jane and Leonard Korman Respiratory institute at Thomas Jefferson University Hospital, 834 Walnut St, Suite 650, 19107 Philadelphia, PA USA

**Keywords:** Lung tumors, SBRT, Rib osteoradionecrosis, Rib fracture, Rib fracture classification

## Abstract

**Background:**

The utilization of stereotactic body radiation therapy (SBRT) is increasing for primary and secondary lung neoplasms. Despite encouraging results, SBRT is associated with an increased risk of osteoradionecrosis-induced rib fracture. We aimed to (1) evaluate potential clinical, demographic, and procedure-related risk factors for rib fractures and (2) describe the radiographic features of post-SBRT rib fractures.

**Methods:**

We retrospectively identified 106 patients who received SBRT between 2015 and 2018 for a primary or metastatic lung tumor with at least 12 months of follow up. Exclusion criteria were incomplete records, previous ipsilateral thoracic radiation, or relevant prior trauma. Computed tomography (CT) images were reviewed to identify and characterize rib fractures. Multivariate logistic regression modeling was employed to determine clinical, demographic, and procedural risk factors (e.g., age, sex, race, medical comorbidities, dosage, and tumor location).

**Results:**

A total of 106 patients with 111 treated tumors met the inclusion criteria, 35 (32%) of whom developed at least one fractured rib (60 total fractured ribs). The highest number of fractured ribs per patient was five. Multivariate regression identified posterolateral tumor location as the only independent risk factor for rib fracture. On CT, fractures showed discontinuity between healing edges in 77% of affected patients.

**Conclusions:**

Nearly one third of patients receiving SBRT for lung tumors experienced rib fractures, 34% of whom experienced pain. Many patients developed multiple fractures. Post-SBRT fractures demonstrated a unique discontinuity between the healing edges of the rib, a distinct feature of post-SBRT rib fractures. The only independent predictor of rib fracture was tumor location along the posterolateral chest wall. Given its increasing frequency of use, describing the risk profile of SBRT is vital to ensure patient safety and adequately inform patient expectations.

## Introduction

Stereotactic body radiation therapy (SBRT) is considered the gold standard therapy for medically inoperable, early-stage non-small cell lung cancer (NSCLC) [1, 2] and an emerging alternative to surgical resection for other lung tumors, including medically operable NSCLC [3] and advanced pulmonary tumors or oligometastases [4–7]. Promising evidence showing positive outcomes has led to rapid increases in utilization of thoracic SBRT, and therefore renewed interest in defining and predicting associated toxicity [8, 9].

Toxicities associated with SBRT include chest wall pain or rib fracture, pneumonitis, esophagitis, and brachial plexopathy, and vary in time of onset. Compared to traditional radiotherapy, SBRT is associated with a higher incidence of toxicity. In particular, the incidence of chest wall pain far exceeds that for traditional radiotherapy, and varies significantly (0.5–46%) [10–16]. Prior studies have demonstrated that both larger total dosages and the volume of bone receiving at least 10 Gy of radiation correlate with increased incidence of rib fracture [11, 17]. However, there remains significant debate regarding the importance demographic and medical risk factors of SBRT-related rib fractures, as well as their radiologic character [13, 14]. The incidence, presentation, and radiographic character of post-SBRT rib fractures are poorly described in the medical literature.

Studies of osteonecrosis following traditional radiotherapy in other cancers correlate dosage and proximity to bone with post-procedural bone fracture [18, 19]. Given the higher dosages delivered by SBRT and immediacy of osseous structures in the chest wall, additional research into the relationship between SBRT and fracture is warranted. Existing cortical bone mapping CT studies following SBRT of affected ribs have shown marked thinning and discontinuous healing patterns, indicating a pathologic process is likely [11, 14]. However, the incidence, presentation, and radiographic character of osteoradionecrosis of the rib are poorly described in the medical literature. As this novel technique for treating and curing lung cancer increases in frequency of use, there is hope that improved understanding of side effects will decrease toxicity and improve patient safety We hypothesize that post-SBRT rib fractures are (1) associated with proximity to the site of radiation, intensity of dosage, and pre-existing comorbidities and (2) have unique radiographic characteristics possibly related to osteoradionecrosis compared to traumatic rib fractures.

## Methods

### Patient selection

Following institutional review board approval, we retrospectively identified patients who underwent SBRT between 2015 and 2018 for both primary and metastatic lung tumors with a minimum 12 month follow up data (mean follow up: 29 months). The need for informed consent was waived by the institutional review board of Thomas Jefferson University and all methods were carried out in accordance with relevant guidelines. Patients without appropriate pre-and post-procedural demographic, medical, and radiographic records were excluded. One patient was excluded for an existing ipsilateral rib fracture, and 24 were excluded for prior radiotherapy to the ipsilateral thorax. In accordance with institutional protocol, computed tomography (CT) was completed prior to treatment and at regular intervals thereafter. Patients with prior ipsilateral rib fractures, ipsilateral chest wall trauma before SBRT, or ipsilateral thoracic radiotherapy for lung, breast, or mediastinal malignancies prior to SBRT were excluded.

### Clinical analysis

Data was gathered by an initial chart review of preoperative consultations and follow up visits, performed by a treating pulmonologist, radiation oncologist, or thoracic surgeon. Documented factors included demographics (age, sex, and self-identified race) and medical comorbidities (chronic obstructive pulmonary disease, diabetes, hypertension, thyroid disease, osteoporosis, and BMI). Procedural factors (tumor staging, tissue of origin, radiation dosage, and fractionation) were also recorded.

### Radiographic analysis

Preoperative CT studies were retrospectively reviewed to determine tumor location, size, and distance from surrounding osseous chest wall structures. Tumor location was classified by lobe, and by location on axial CT section. Tumor location was adapted from Kim et al. and Liebsch et al. [14, 20]. On transverse section, along the midline anterior-posterior axis, the length of the rib was divided equally into 5 sections (36^o^ each), corresponding to anterior, anterolateral, lateral, posterolateral, and posterior. Tumors along the medial region of the pleura, or more than 20 mm from the chest wall were classified as either central or hilar, depending on location. The first author then reviewed postoperative CT images, ordered primarily to monitor for disease progression, to identify evidence of post-radiation rib fracture, including discontinuous or healing lesions. Identified fractures were further classified by the degree of radiographically-observable discontinuity between the healing edges, modified from the classification described by Kim et al. [14]. Grade 1 fractures show discontinuous healing edges displaced less than half the diameter of the rib. Grade 2 fractures were discontinuous with healing edges displaced greater than half the diameter of the rib. Continuous or hairline fractures were classified as such and counted separately. For patients with rib fractures, CT imaging of the contralateral side was reviewed to identify and co-occurring rib fractures to the non-irradiated hemithorax.

### Stereotactic body radiation therapy

Planning target volume (PTV) was determined via four-dimensional competent tomography. Internal tumor motion was accounted for through the entire respiratory cycle, and a 5 mm expansion was added to arrive at the final PTV (Agility™, Elekta; Stockholm, Sweden). Treatments were delivered every other day between 3 and 12 fractions. The total dosage delivered ranged from 48 to 60 Gy. The most common fraction schedule was 60 Gy delivered in 5 fractions.

### Statistical analysis

Multivariate analysis for post-radiation rib fracture risk factors was performed using logistic regression with all collected variables. The regression model was constructed by sequentially adding demographic, clinical, and procedural variables and monitoring the strength of association. The reported odds ratios were determined from the final model. The distance between tumors and nearby osseous structures was dichotomized to improve analysis, and provide a more practical clinical marker of fracture risk. A cutoff of 12 mm was chosen arbitrarily to identify tumors within close proximity of the chest wall. Identified rib fractures were then examined separately. All analyses were performed using SPSS version 26.0 (Chicago, IL).

## Results

A total of 106 patients with 111 (five patients had two tumors, treated simultaneously) tumors met the inclusion criteria, 35 of which showed evidence of post-SBRT rib fractures, a total of 60 fractured ribs. The median follow-up time was 25 months. Patient demographics are shown in Table 1. Posterolateral tumor location was the only factor independently associated with increased risk of rib fracture (OR: 4.92 [1.51–16.03]; Table 2). Neither age, sex, BMI, nor race were associated with fracture incidence (p = 0.376, 0.153, 0.850, and 0.999, respectively). Hypertension was correlated incidence (p = 0.009; Table 1), but not independently associated (OR: 2.66 [0.98–7.22]; Table 2) with increased fracture risk. Similarly, anterolateral and lateral tumor location were correlated (p = 0.041 and 0.033, respectively) but not independently associated (OR: 4.06 [0.91–18.05] and 1.85 [0.70–4.91], respectively) with rib fracture. Of the 35 patients who experienced rib fractures, 35% were treated for tumors within 12 cm of the chest wall (Table 1). However, tumor distance under 12 cm was not an independent predictor of rib fracture. Tumor size and radiation dosage showed no association with rib fracture (p = 0.218 and 0.259, respectively).

**Table 1:** Patient demographics, comorbidities, and procedural details

		No Fracture		Fracture		*p* value
		n	%	n	%	
		76	68	35	32	
Sex						
	Male	35	46	13	37	0.153
	Female	41	54	22	63	
Self-Identified Race						
	Asian	5	6	2	6	0.999
	Black	15	20	5	14	0.999
	Hispanic/Latino	2	2	2	6	0.999
	White	55	72	26	74	0.999
Comorbid Conditions						
	Diabetes	9	12	3	8	0.227
	Hypertension	49	64	29	83	0.009
	Thyroid Disease	13	17	8	23	0.075
	Osteoporosis	11	14	4	11	0.531
	COPD	23	30	12	34	0.435
Tumor Region						
	Anterior	7	9	1	3	0.960
	Anterolateral	3	4	4	11	0.041
	Lateral	11	14	10	29	0.033
	Posterolateral	5	6	9	26	0.021
	Posterior	10	13	7	20	0.070
	Central	36	47	4	11	0.809
	Hilar	4	5	0	0	0.999
Distance to Closest Rib						
	Greater than 12 mm	46	61	6	17	
	Less than 12 mm	30	39	29	83	0.978
Lobe Affected						
	Upper	45	60	20	57	
	Middle	5	6	0	0	
	Lower	26	34	15	43	
	Right	49	64	22	63	
	Left	27	36	13	37	
Tumor Staging						
	T1N0M0	51	67	28	80	
	T1N0M1	1	1	0	0	
	T2N0M0	8	11	3	8	
	T2N1M0	4	5	0	0	
	T2N3M0	1	1	0	0	
	T2N0M1	2	3	1	3	
	T3N0M0	4	5	0	0	
	T3N4M0	1	1	0	0	
	2^o^ Metastasis	4	5	3	8	
		**Mean**		**Mean**		***p value***
Age		72.6		71.7		0.376
BMI		26.8		25.7		0.850
Tumor Diameter (mm)		20.3		19.9		0.218
Cumulative Dose (Gy) ^a^		54.7		56.7		0.259

**Table 2:** Multivariate analysis of demographic and procedural risk factors in fracture vs. non-fracture groups

		Value	95% Confidence Interval	
			Lower	Upper
Female Sex		0.692	0.305	1.573
Self-Identified Race				
	Asian	0.861	0.159	4.669
	Black	0.678	0.225	2.041
	Hispanic/Latino	2.242	0.303	16.610
	White	1.103	0.444	2.739
Comorbidities				
	Diabetes	0.698	0.177	2.754
	Hypertension	2.663	0.983	7.216
	Thyroid Disease	1.436	0.534	3.862
	Osteoporosis	0.762	0.225	2.587
	COPD	1.202	0.513	2.820
Tumor Region				
	Anterior	0.290	0.034	2.452
	Anterolateral	4.056	0.911	18.051
	Lateral	1.846	0.695	4.905
	Posterolateral	4.915	1.507	16.028
	Posterior	1.477	0.591	4.205
	Central	0.218	0.081	0.585

Fractures were noted in every rib except the twelfth, and most commonly affected ribs 2, 3,4, 7, 8, and 9 (Table 3). 47% of fractures occurred at the rib closest to the treated tumor, and a further 40% occurred at the second closest rib. The remaining 13% occurred two or more ribs removed from the closest rib (Table 3). Fractures were first noted, on average, 22 months following the completion of SBRT. Fractures were most common along the lateral regions of the chest wall, and least common in the anterior region of the chest wall related to tumors along the costal cartilage (Table 1). Roughly half (54%) of post-radiation rib fractures were limited to a single rib, while two ribs were affected in 29% of cases, and 3 to five were impacted in 17% of cases (Table 3). Four patients reported post-procedure chest wall trauma, three of whom developed rib fractures, resulting in a total of five fractured ribs. The majority (77%) of rib fractures showed a characteristic discontinuity between the healing edges of the fractured rib, 48% showed minor discontinuity (Gr1), while 29% showed displacement greater than half the diameter of the rib (Gr2). Pain associated with rib fracture was only reported in 34% of affected subjects, while 89% of subjects reported no trauma prior to fracture incidence (Table 3). Most (59%) fractures were noted on the radiologist’s report during the full duration of care, but only 21% were specifically mentioned at initial appearance. The remainder (41%) were never mentioned in a radiologist’s report, either at first appearance or in subsequent routine follow-up imaging. It should be noted that the studies reviewed were ordered to monitor for cancer progression, and not specifically to identify rib fracture. Only three of 35 affected patients had evidence of rib fractures to the contralateral (non-irradiated) side. Two previously underwent SBRT to the contralateral hemithorax for lung tumors, and the other the third had a pre-existing healed fracture present on prior imaging.

**Table 3:** Characteristics of observed rib fractures

		n = 35		
		**Mean**		**Stdev**
Interval ^a^ (months)		21.7		11.8
Distance from tumor (mm)		13		17
		**n = 60**		**%**
Rib number				
	1	1		2
	2	8		13
	3	8		13
	4	11		18
	5	4		7
	6	5		8
	7	6		10
	8	8		13
	9	6		10
	10	2		3
	11	1		2
	12	0		0
	Total	60		100
Number of Ribs Fractured				
	1	19		54
	2	10		29
	3	4		11
	4	1		3
	5	1		3
Fractured Rib Relation to Tumor				
	Closest to tumor	28		47
	1 rib removed from tumor	24		40
	2 + ribs removed from tumor	8		13
	Total	60		100
Visible Discontinuity				
	Gr1	17		48
	Gr2	10		29
	Total	27		77
	No Discontinuity	8		23
		**n = 35**		**%**
Number of Ribs Fractured				
	1	19		54
	2	10		29
	3	4		11
	4	1		3
	5	1		3
Associated Chest Wall Pain		12		34
Documented Trauma		3		8
Date Mentioned on Radiologist’s Report ^b^				
	At first incidence	7		21
	Retrospectively ^c^	13		38
	Not Reported	14		41

^a^ Interval from procedure date to fracture incidence

^b^ Reports available for 34 of 35 subjects

^c^ Fracture mentioned retrospectively in studies done after the initial incidence

## Discussion

Rib fractures are a significant toxicity following SBRT for both metastatic and primary lung tumors. Fracture-associated chest wall pain may interfere with respiration in patients with advanced respiratory illness, while corresponding instability may increase the risk of significant injury in future cases of thoracic trauma. In this study, the incidence of post-radiation rib fracture was 32% and was independently associated with posterolateral tumor location. The overwhelming majority (87%) of fractures occurred at the closest or second-closest rib to the treated tumor, suggesting an association between treatment and fracture. The majority of fractures were asymptomatic, but 34% of patients with fractures experienced chest wall pain. Post-SBRT rib fractures showed radiographically distinct characteristics with discontinuity between the healing edges. Such findings were largely underreported by reviewing radiologists on routine follow up imaging.

Posterolateral tumor location was the only independent predictor of post-radiation rib fracture, while anterolateral and lateral location were also correlated with fracture. Conversely, central tumor location appeared to have a protective effect against rib fractures. Though univariate analysis showed no significant association, multivariate modeling showed a lower risk of rib fracture in centrally-located tumors, further evidence that tumor location impacts post-SBRT fractures. Two prior studies demonstrated a similar pattern, where lateral treatment sites were more prone to rib fracture than anterior or posterior lesions [14, 21]. The association between treatment site and rib fracture is likely multifactorial. Biomechanical studies suggest that forces applied to the chest wall are distributed across the body of the rib to the anterolateral, lateral, and posterolateral regions of the rib, depending on the direction of force [20]. Osteoradionecrosis at sites already prone to rib fracture could thus explain the disproportionate incidence observed in the present study. Further, the anterior and posterior segments of ribs are stabilized somewhat by surrounding structures. Anteriorly, most lesions abut the costal cartilage, whose response to SBRT has not been reported. Posteriorly, the intrinsic back muscles and structures of the spine may provide increased stability and shielding to the ribs, decreasing radiation injury to osseous structures. While few subjects reported trauma, this biased distribution may indicate that mechanical triggers still play a role in rib fracture. Forces from subtle stressors, such as coughing, sneezing, or minor external pressure, when repeated overtime and directed disproportionately to the posterolateral curvature of the rib may lead to fractures.

The relationship between post-treatment fractures and medical comorbidities was less significant. There is debate in the existing literature over the impact of medical comorbidity and chest wall toxicity; previous studies indicate that sex, race, and diabetes history may all be risk factors [13, 14]. While hypertension did correlate with an increased rate of fracture in the present study, it was not an independently significant predictor. Such disagreement suggests that comorbidity may not directly influence fracture risk. Age was similarly non-predictive of fracture risk. The relative independence from medical comorbidities may have important consequences with the increasing utilization of SBRT [3, 5]. Expanding the potential patient population to include typically healthier surgical candidates may not reduce fracture incidence. As younger, healthier patients receive SBRT at increasing rates, the clinical significance of chronic osteonecrotic fractures may become more pronounced. Still, further investigation including more granular endocrine, hematologic, or nutritional studies may be warranted to pinpoint at-risk patients in an expanding patient population.

Unlike traumatic fractures, post-SBRT rib fractures show distinct radiographic discontinuity between healing edges. The majority (77%) of SBRT-induced rib fractures observed in the present study showed discontinuity, a significant number of which (29%) were severe (Gr2). Previous studies have shown similar rates of discontinuity, but the pathogenesis and consequences of these features remain undefined [13, 14]. Our study does not address the specific mechanism of rib fracture after SBRT. Hypotheses could include bone demineralization or osteoradionecrosis. The occurrence of fractures 22 months after SBRT, on average, would argue against trauma during the treatment sessions. The discontinuity of the fracture healing does suggest an alteration in osteoblast progenitor cells. Cortical bone thinning is associated SBRT-induced fractures, but its specific role in fracture discontinuity in unknown [11]. The fracture frequency in our population was similar for patients with or without COPD suggesting that exposure to tobacco or other risks for osteoporosis were not necessarily important factors. Future research could include T1-weighted MRI and PET/CT studies, as well as pathology at autopsy, to evaluate the pathogenesis of post-SBRT fractures and discontinuity. Further, Chipko et al. suggested that severe discontinuity may involve fibrosis of surrounding soft tissue and lead to higher short-term pain [13]. The potential for discontinuous fractures to remain non-united or malunited may thus have important long-term clinical consequences for the management of affected patients.

The role of chest wall trauma in the pathogenesis of post-SBRT rib fractures also warrants further investigation. Relevant trauma was documented for four patients in the present study, three of whom developed rib fractures. However, the true influence of trauma on post-radiation fractures is difficult to accurately quantify without a more consistent definition and evaluation of trauma. In the present study, trauma was patient reported. Data was therefore subject to biases in reporting and differing patient thresholds for what constitutes significant trauma. Given the potential importance of major and minor forces, more granular data is needed. Future studies may benefit from prospectively designed criteria for trauma which accounts for the site, mechanism, and force of impact.

Despite radiographically distinct features, the prevalence of post-SBRT rib fractures may remain underreported. Prior broader studies by Lagerwaard et al., Ricardi et al., and Asai et al. reported low rates of rib fracture (4.1%, 1.8%, and 24%, respectively) despite large sample sizes [15–17]. The majority of fractures in the present study were asymptomatic (66%) and nontraumatic (89%), suggesting that factures may not be identified without regular follow up CT imaging. Given that follow up imaging was focused on monitoring cancer progression, fractures were also not always included in the radiologist’s report. These barriers to identification may explain why earlier studies with broader aims have reported such low incidence of post-SBRT rib fracture, and suggest the true incidence is likely higher, as reported in the present study (32%) and other recent, more focused investigations [13, 14].

We included four subjects who experienced post-SBRT trauma and subsequently developed rib fractures in the present study. This included one subject who was hospitalized for a mechanical fall, and rib fracture was identified on admission. Three other subjects had suspected recurrent falls after SBRT secondary to other medical conditions. Fractures in all three subjects were identified incidentally and were not directly related to a specific event. It is difficult to determine to what degree SBRT contributed to traumatic fractures. Even in traumatic falls, radiotherapy may predispose patients to rib fractures which otherwise may not have occurred. We thus included these four traumatic cases to fully describe the total incidence of rib fracture after SBRT.

The principal strengths of the present study include the large patient population, consistent radiological follow up with CT imaging, and non-exclusion of metastatic or late-stage lung tumors. Given the retrospective study design, follow-up bias may have influenced the high incidence of rib fracture. Given the relatively low symptom rate (34%) of fractured ribs, though, we believe our conclusions remain valid. The minimum period of follow up for inclusion in the present in our study was 12 months. However, the average follow-up (29 months) was greater than the average interval to fracture identification (22 months). Therefore, it is possible that the shorter minimum follow-up period meant that some subjects who may have gone on to develop rib fracture were missed. Further studies with longer follow up are still warranted to catch later-onset or later-detected rib fractures. Analysis of the longer-term progression of rib fracture and osteoradionecrosis is also warranted given the possibility of non- or malunion. Future research could also include more targeted bone marrow, endocrine, and soft tissue studies, as well as more descriptive radiotherapy parameters, to better characterize the risk and pathogenesis of post-SBRT rib fractures. Lastly, while the present study analyzed a significant number of rib fractures, the sample size of some smaller subgroups is limited. Future research could specifically target such subgroups, such as tumor staging, to analyze specific risk factors with greater statistical power.

## Conclusions

While the utilization of SBRT continues to expand, understanding the risk factors and potential sequelae of rib fracture is vital for properly informing patient expectations and maintaining safety. Rib fractures following SBRT showed distinct radiologic discontinuity between healing edges and tumor location along the posterolateral chest wall was an independent predictor of rib fracture. The incidence of fracture was 32%, while 34% of fractures presented with chest wall pain. Rib fractures represent a significant toxicity which may lead to chest wall instability fracture-associated interference in respiration. We conclude that all patients with lung tumors along the posterolateral chest wall, especially those within a 12 mm radius, be advised of the risk of rib fracture prior to undergoing SBRT.

## Data Availability

The datasets used and/or analyzed during the current study are available from the corresponding author on reasonable request.
